# Postoperative mortality after cancer surgery in octogenarians and nonagenarians: results from a series of 5,390 patients

**DOI:** 10.1186/1477-7819-3-71

**Published:** 2005-11-09

**Authors:** Ronald AM Damhuis, Claudia JC Meurs, Willem S Meijer

**Affiliations:** 1 Rotterdam Cancer Registry, Rotterdam, The Netherlands; 2 Medical Center Rijnmond-Zuid, Department of Surgery, P.O. Box 9119, 3007 AC Rotterdam, The Netherlands

## Abstract

**Background:**

To support decisions about surgical treatment of elderly patients with cancer, population-based estimates of postoperative mortality (POM) rates are required.

**Methods:**

Electronic records from the Rotterdam Cancer Registry were retrieved for octogenarians and nonagenarians who underwent resection in the period 1987–2000. POM was defined as death within 30 days of resection and both elective and emergency operations were included.

**Results:**

In a series of 5.390 operated patients aged 80 years and older, POM rates were 0.5% for breast cancer, 1.7% for endometrial cancer and 4.2% for renal cancer. For patients with colorectal cancer, POM increased from 8% for the age group 80–84 to 13% for those 85–89 to 20% in nonagenarians. For stomach cancer, the respective figures were 11%, 20% and 44%.

**Conclusion:**

These results show that resections can be performed at acceptable risk in selected elderly patients with cancer.

## Background

As a result of ageing of the general population, the proportion of elderly patients with cancer is increasing within Europe. In the Netherlands, 14% of male patients and 17% of female patients are 80 years or older at diagnosis [1]. In the elderly, relative survival is generally worse than in younger patients [2], mainly due to a more advanced stage at diagnosis or due to less extensive treatment. Especially surgery is withheld out of concern for postoperative morbidity and mortality. Many studies, however, suggest that surgical treatment can be performed at acceptable risk and with good results. These reports usually come from specialized centres with selected series and may be too optimistic due to reporting and publication bias. To support decisions about the option of surgical treatment in a general situation, we studied postoperative mortality rates using data from a population-based cancer registry.

## Methods

Information on octogenarians and nonagenarians who underwent resection for cancer in the period 1987–2000 was retrieved from the Rotterdam Cancer Registry. The Rotterdam Cancer Registry is operational since 1982 and now covers a region with one university hospital, 15 general hospitals and 2.3 million inhabitants. Trained coding clerks collect information on tumour site and morphology, tumour stage and type of treatment. The clinical record is assessed at least 3 months after diagnosis and notification, thus enabling the evaluation of postoperative mortality, which was defined as death within 30 days of operation. Due to privacy regulations death certificates are not available, thus hampering evaluation of long-term survival.

This study comprises all consecutive patients who underwent primary resection of the tumour, irrespective of the type of surgery, whether being elective, emergency, curative or palliative. To avoid chance findings, the study was restricted to types of cancer with more than 100 patients operated. Subgroup analysis by age was only performed for types of cancer with more than 10 postoperative deaths per subgroup. Postoperative mortality rates were tabulated and differences between subgroups were evaluated with chi-square test.

## Results

This study comprises 5,390 patients who underwent resectional surgery between 1987 and 2000. Postoperative mortality increased with age from 5.4% in patients aged 80 to 84 years, to 9.1% in patients aged 85 to 89 years and to 14.4% in nonagenarians (Table 1). In more recent years, a small decrease in postoperative mortality was observed, from 7.7% in the period 1987–1993 to 6.5% in the period 1994–2000 (p = 0.07).

**Table 1:** Postoperative mortality (POM) in octogenarians and nonagenarians who underwent resectional surgery for cancer

		**Postoperative mortality**		
		**No. of patients**	**No. of deaths**	**%**

**Sex**	Male	1385	184	13.3
	Female	4005	196	4.9
**Age (years)**	80–84	3513	190	5.4
	85–89	1503	136	9.1
	90+	374	54	14.4
**Period**	1987–1993	2415	187	7.7
	1994–2000	2975	193	6.5
**Type of cancer**	Colorectal	2765	293	10.6
	Stomach	424	67	15.8
	Breast	1731	9	0.5
	Endometrial	350	6	1.7
	Kidney	120	5	4.2

Colorectal cancer and breast cancer were the most common types of cancer with postoperative mortality rates of 10.6% and 0.5%, respectively. Postoperative mortality was highest for stomach cancer with 15.8%. For endometrial cancer and kidney cancer, postoperative mortality rates were 1.7% and 4.2%, respectively.

For colorectal cancer, postoperative mortality rates increased from 8% in patients aged 80 to 84 years, to 13% in patients aged 85 to 89 years and to 20% in nonagenarians (p < 0.001) (Table 2). For stomach cancer the respective figures were 11%, 20% and 44% (p < 0.001).

**Table 2:** Postoperative mortality for octogenarians and nonagenarians with colorectal or stomach cancer.

Type of cancer	80–84 years		85–89 years		90+ years		
	n	POM (%)	n	POM (%)	n	POM (%)	p-value

Colorectal	1731	8	837	13	197	20	<0.001
Stomach	293	11	99	20	32	44	<0.001

## Discussion

Our results show that resectional surgery can be performed at acceptable risk in elderly patients with cancer. It is unknown whether these results can be generalised to other regions, time periods or health care systems. Population-based studies tend to report higher operative mortality rates than studies from single institutions. Selection criteria are presumably different from those in trial series and publication bias hampers honest comparison. Even between population-based studies, selection criteria and definitions may differ. For example, a study from the US [3] analysed operative mortality in patients aged 85 years and older and reported on gastrectomy (16%), colectomy (7%) and nephrectomy (5%), but only included elective operations. After emergency surgery, postoperative death may not be attributable to the resection itself because some patients would also have died after alternative treatment. A second difference is that the US study reported on in-hospital mortality, which produces higher rates than the 30-day definition.

Age-specific reference figures are essential for clinical decision-making but calendar age serves as a poor substitute for biological age, which in itself is difficult to define or determine [4]. Preoperative assessment of the operative risk in geriatric patients is considered very important but current scoring methods provide little assistance [5]. As a consequence, treatment decisions in the elderly cannot be based on general guidelines but will rather require tailored plans that need to be discussed with the patient and his or her family. In some cases, surgery may need to be postponed for the treatment of concomitant disease.

Apart from operative mortality, the remaining quality of life and the life expectancy should be taken into consideration. Non-fatal complications and lengthy hospital stays should not be taken too lightly and may severely impair daily living. The median life expectancy for men and women aged 90 years, is 3.3 and 4.1 years, respectively. The actual life expectancy, however, is difficult to predict and little is known about the course of cancer after suboptimal treatment. Especially in the elderly, preoperative assessment of the extent of disease is crucial to avoid senseless palliative surgery.

For breast cancer, studies have shown that primary hormonal treatment is inferior to direct surgery [6], which is understandable given the low operative risk. For endometrial cancer, the operative risk is less than 2% and alternative treatments should be reserved for patients with severe comorbidity. For kidney cancer, nephrectomy should be considered when urinary function is acceptable, but cancer progression may be slow and a wait-and-see policy is a serious option in patients with smaller lesions. For abdominal surgery, the operative risk is substantial but this risk may still be worthwhile given the absence of other curative options. Whether an operative risk of 44% in nonagenarians with stomach cancer is ethically acceptable, is up to discussion. However, when faced with a guarantee of progressive cancer and no alternatives for cure, patients are willing to accept extremely high-risks [7] that may even seem unacceptable to their physician. For abdominal tumours, emergency presentation is more common in the elderly and obstruction may lead to a quick death in case of non-surgical treatment. Laparoscopic interventions may, however, avert major surgery.

## Conclusion

In the near future, the number of elderly patients with cancer will continue to rise due to the ageing of the general population. Judging from the current findings, surgery should not be withheld because of the postoperative mortality but suboptimal or palliative treatment may be necessary in patients with poor physical or mental function. To enable informed decision-making, both patients and clinicians need information on the risks of surgical treatment. This information should be retrieved from population based studies since we cannot rely on the memory of individual clinicians, as is shown by the nine deaths after surgery for breast cancer in a consecutive series of 1731 patients in 15 hospitals over a period of 14 years. Finally, we want to stress the importance of studies incorporating comprehensive geriatric assessment that may provide us with better estimates of the operative risk in the future [8].

## Competing interests

The author(s) declare that they have no competing interests.

## Authors' contributions

**RD **Study management, literature review, preparation of manuscript

**CM **Study design, statistical analysis

**WM **Study material contribution, interpretation of data, critical revision of manuscript
